# Monitoring of Rubber Belt Material Performance and Damage

**DOI:** 10.3390/ma17030765

**Published:** 2024-02-05

**Authors:** Tomasz Ryba, Damian Bzinkowski, Zbigniew Siemiątkowski, Miroslaw Rucki, Sylwester Stawarz, Jacek Caban, Waldemar Samociuk

**Affiliations:** 1 Faculty of Mechanical Engineering, Casimir Pulaski Radom University, Stasieckiego Str. 54, 26-600 Radom, Poland; tomasz.ryba@itee.lukasiewicz.gov.pl (T.R.); damianbzinkowski@gmail.com (D.B.); z.siemiatkowski@uthrad.pl (Z.S.); stawarz@uthrad.pl (S.S.); 2 Institute of Mechanical Science, Vilnius Gediminas Technical University, Jono Basanaviciaus Str. 28, LT-03224 Vilnius, Lithuania; 3 Faculty of Mechanical Engineering, Lublin University of Technology, Nadbystrzycka Str. 36, 20-618 Lublin, Poland; 4 Faculty of Production Engineering, University of Life Sciences in Lublin, Gleboka Str. 28, 20-612 Lublin, Poland

**Keywords:** rubber, damage identification, monitoring, predictive maintenance, belt conveyor

## Abstract

Conveyors play a very important role in modern manufacturing processes, and one of the most popular types is the belt conveyor. The main elements of a conveyor include a conveyor belt, roller sets, a supporting frame and a drive and control system. The reliable operation of the conveyor depends on the strength and durability of individual elements (especially the belt). Conveyor belts are made from various materials and have received a lot of attention in the scientific and research community. This article presents tests of the strength of the rubber belt material and its damage under load. The belt consists of two internal layers covered with a PVC coating on the outside, and the nominal belt thickness was 2 mm. In the experiment, various configurations of longitudinal and transverse damage were verified, and statistical methods were used to analyze the results. The obtained test results provided a new understanding of the propagation of conveyor belt damage and helped to improve the strain gauge-based monitoring system.

## 1. Introduction

One of the most commonly used means of continuous transport in industry is a belt conveyor. Continuous transport devices can be used to easily move materials that exceed human capabilities in terms of weight and height [1,2]. Depending on the industry in which the belt conveyor is used, it must meet appropriate criteria and specific requirements, such as sterility in the food industry or high durability in mining or construction applications. The issues of testing the supporting structure of conveyors and their moving units are presented in [3,4,5,6,7], among others.

Indeed, there is an increasing number of publications devoted to monitoring systems for belt conveyors. Even though the number of lethal accidents is decreasing, there are still a large number of failures that could be predicted if monitored properly [8]. Among the large variety of the monitoring devices, the following can be noted: visual systems [9], including laser-based systems [10] and multispectral image acquisition systems [11,12,13], acoustic emission systems [14,15,16,17,18,19], ultrasound detectors [20], thermovisual systems able to detect the elevated temperature of the intensely worn belt surface [21], strain gauge measurement systems [22], and others [23,24,25]. Most of the proposed solutions require the introduction or adaptation of expensive devices and exhibit limited repeatability and accuracy of the measurement. The proposed monitoring system based on strain gauges is cheap and simple, is easily replaceable and adjustable, and has a high level of ability to monitor the working belt in real time.

The material of the rubber belt in industrial conveyors is responsible for the correct transportation of components within the factory and also for most of its failures. For this reason, it is necessary not only to measure the strength and resistance to degradation of the belt material, but also to monitor its wear and performance. In this way, it will be possible to detect emerging damage and prevent failures, introducing so-called ‘predictive maintenance’ [26,27,28,29].

The reliability of transport systems requires the use of high-quality (relative to the price) conveyor belts. During the operation of machines, damage occurs and must be removed as quickly as possible. The useful life of the tapes depends on their tensile strength. Tensile strength depends on the type and size of damage. In addition to online process monitoring [30], actions are recommended (e.g., risk analysis [31]) to extend the time required to repair the damaged device. Knowledge of the properties of conveyor belts is necessary to select process parameters. It is important to establish how damage (type and size of damage) affects the reliability of the device.

This article presents tests of conveyor belt damage using an innovative belt condition monitoring system and strength tests for various cases of belt damage. The description of the tests performed is presented in Section 2 (Materials and Methods), while the results and their analysis are included in Section 3 (Results and Discussion). Section 4 (Conclusions) contains a short summary and main conclusions from the research conducted.

## 2. Materials and Methods

Our research on rubber belt material included investigations on its strength and damage formation under loads, but also the ability of a strain gauge-based monitoring system to identify appearing damage.

The type of the belt used in the tests was EDV08PB-AS 2.0 produced by Enitra Sp. z o.o. (Wałbrzych, Poland). It had two inner layers covered with the PVC coating on the outer sides, and the nominal thickness of the belt was 2 mm. The belt material’s rigidity allowed for applications with rollers with diameters of no less than 30 mm.

### 2.1. Monitoring System Based on Strain Gauges

**Figure 1:**
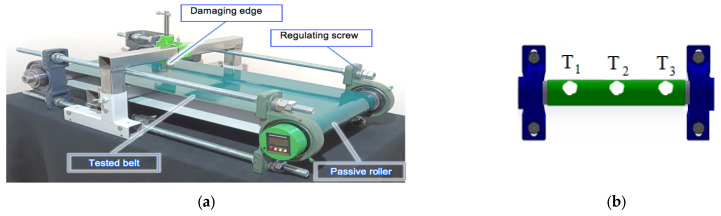
The test rig for conveyor belt damage monitoring: (**a**) overall view; (**b**) position of the strain gauges T1, T2, and T3 on the conveyor’s passive roller.

The work used a newly developed monitoring system, which is the subject of a patent application. Its main sensing element was a strain gauge CP152NS (IEE S.A., Bissen, Luxembourg), controlled by a data processing system, and described in detail elsewhere [22]. Three identical strain gauges denoted as T1, T2, and T3 were placed on the surface of the conveyor’s passive roller in the manner shown in Figure 1. During the conveyor operation, the belt generated pressure on the roller which was registered by the strain gauges and further processed.

The test rig consisted of two rollers, passive and active ones, and the rubber belt between them. The system made it possible to regulate the rollers’ position to ensure the necessary tension of the belt. The data processing system was placed inside the passive roller. It communicated with an external computer using a Bluetooth wireless connection. The dedicated program was prepared specially for the data record and further analysis. An example of the registered data was published along with the initial results of machine learning tests for the load identification [32].

The strain gauges underwent calibration according to a specially designed procedure [33]. Before the tests, the initial setting of the rollers was performed in order to ensure equal pressure on both strain gauges, T1 and T3. Indications of the central gauge T2 were omitted in these proceedings, since its indications were dependent only on the inner stresses inside the rubber belt material.

### 2.2. The Rubber Belt Damage Monitoring

**Figure 2:**
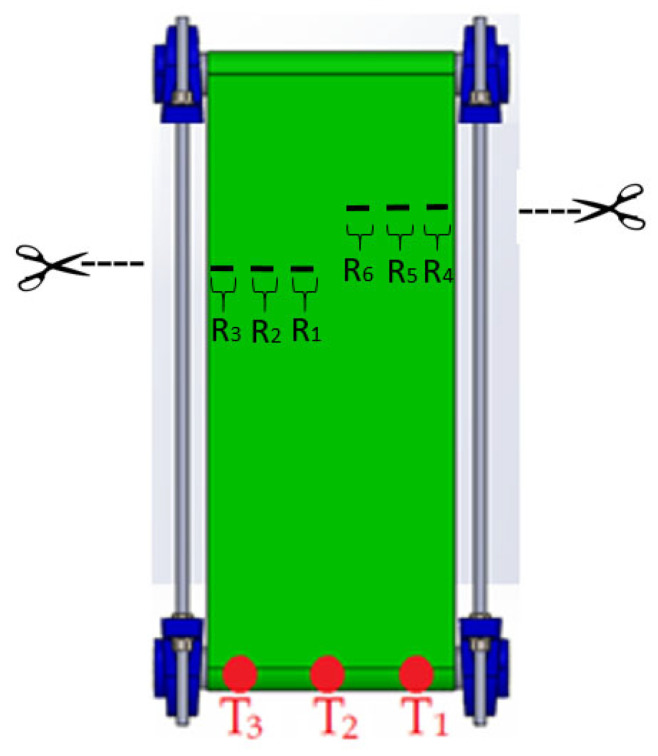
Sequence of cuts to the belt during the tests.

Since the damage to the belt was irreversible, the monitoring of the damaged belt was performed after sequential addition of more damage. The sequence of cuts was denoted as R1, R2, and R3 on the right side of the belt and R4, R5, R6 on the left side, respectively, as shown schematically in Figure 2.

Each individual damage was exerted perpendicularly to the belt movement direction, and its length was 50 mm. After adding each subsequent cut, the conveyor worked for ca. 10 min at a roller rotational speed *f*_1_ = 400 rpm corresponding with a belt linear speed of *v*_1_ = 0.5 m/s. During this time, 10,000 indications of the strain gauges were sampled and registered. The as-obtained results were compared with those obtained under similar work conditions of the belt without damage and loads.

Two extreme situations, when the belt was broken completely or slid off the strain gauge, were not considered. It is obvious that in these situations, the gauge would stop receiving any pressure and start indication failure without any need for damage analysis.

### 2.3. The Rubber Belt Strength

During the research, two hypotheses were adopted regarding the durability of textile conveyor belts:

**H1.**  *The strength of textile conveyor belts differs for longitudinal loads and for transverse loads*.

**H2.**  *The durability of textile conveyor belts depends on the type (direction) and size of damage*.

#### 2.3.1. Experiment Plan

The experiment plan involved comparing the strength of textile conveyor belts for longitudinal and transverse loads (factor 1). The tensile test of the samples cut off from the rubber belt in the longitudinal and perpendicular directions was performed as follows.

Textile conveyor belts were subjected to damage, either transverse and longitudinal (factor 2), and the following tests were performed:

- Tensile tests of the samples with longitudinal damage;
- Tensile tests of the samples with perpendicular damage.

Transverse damage was exerted over a length of 10 mm. The longitudinal damage had 3 different lengths and 3 types of damage depth (these were not the same cuts as those presented in Section 2.2).

The research was carried out in accordance with a 2-factor full experimental design. Table 1 shows the grouping of samples.

#### 2.3.2. Methodology

General characteristics of the study. The tensile testing of textile conveyor belts involved uniaxial deformation of a sample of specific dimensions at a constant speed and measuring the force depending on the deformation:

F = f(ε),(1)

where ε is the strain, as an independent variable, and F is the force depending on deformation.

From the obtained graphical relationship, the physical properties were calculated:

(a) Tensile strength;
(b) Elongation at breaking-point;
(c) Tensile modulus.

Strength tests were carried out for conveyor belts in accordance with the EN ISO 283:2023 standard: Textile conveyor belts—Full thickness tensile strength, elongation at break and elongation at the reference load—Test method [34]. The cited International Standard presents a test method used to determine the tensile strength through the thickness in the longitudinal direction and the elongation at the reference force and breaking point of conveyor belts with a textile matrix.

According to the cited standard, the following terms and definitions were used:

Tensile strength (TS)—greatest measured force during the tensile test divided by the width of the test piece (N/mm);

Nominal tensile strength (NTS)—specified minimum value of the tensile strength (N/mm);

Reference force, reference load (RF)—one-tenth of the nominal tensile strength in the longitudinal direction multiplied by the width of the test piece in mm;

Elongation at break (EB)—elongation at the greatest force (load);

Elongation at the reference force (load) (ERF)—elongation at the reference force (load) in the longitudinal direction;

Strain—ε, increase in length per unit original length of the gauge.

The elongation was determined using an extensometer. All strain values were calculated using the following equation:

ε = ∆L_0_/L_0_, (2)

where ε is the value of a given strain (expressed as a percentage); L_0_ is the measurement length of the tested sample (expressed in millimeters); and ΔL_0_ is the increase in sample length between the gauge marks (expressed in millimeters).

The analysis of the results was extended, and the tensile modulus was determined in accordance with the ISO 527-1:2012(E) standard [35].

The tensile modulus, Et, was calculated as the slope of the stress/strain curve σ(ε) in the strain interval between ε1 = 0.05% and ε2 = 0.25%:

Et = (σ2 − σ1)/(ε2 − ε1),(3)

A test piece, cut from the full thickness of the conveyor belt, was extended under specified conditions using a tensile testing machine, until the rupture of the test piece occurs. The testing machine stretched the test sample continuously at a constant speed of (100 + 10) mm/min.

**Figure 3:**
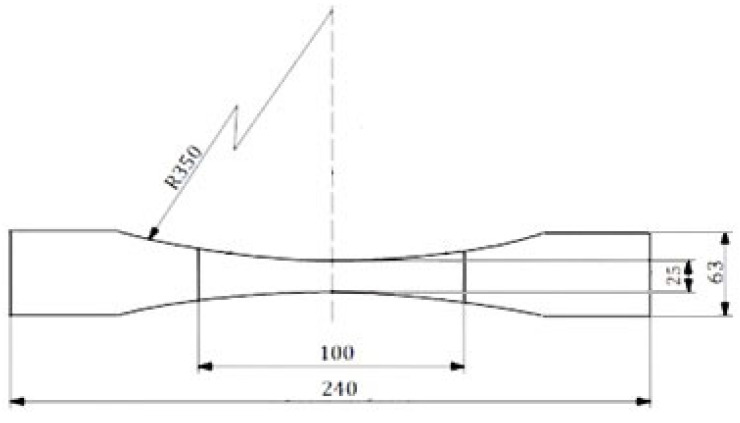
Type C test piece (in mm).

The shape and dimensions of the test piece were measured in accordance with Figure (Figure 3 in standard EN ISO 283:2023).

Static tensile tests were carried out using a ZWICK Z100 (ZwickRoell GmbH, Ulm, Germany) testing machine. A 10 kN head was used in the tests (with force measurement accuracy from 0.2 kN), and the deformation was measured using a tentacle macro extensometer.

Conditioning. The samples were kept in the room where the testing machine was located. During the measurements, the temperature in the room was 24 °C and the humidity was 45%.

**Figure 4:**
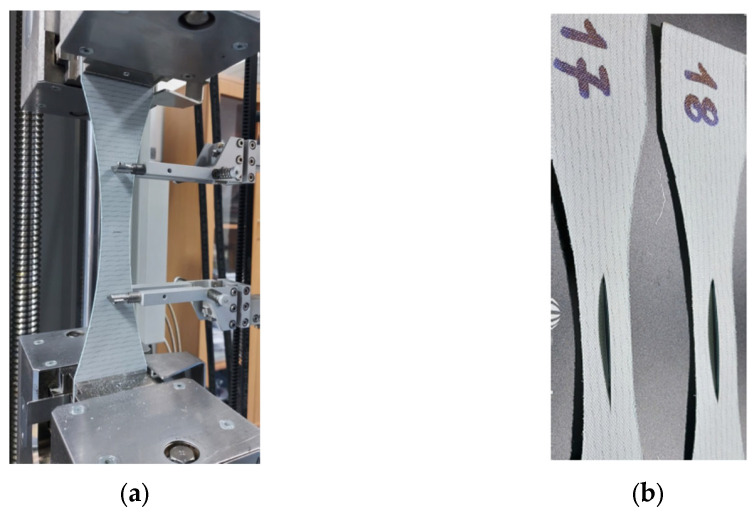
Samples of the belt material: (**a**) example of the sample with perpendicular damage during the tensile test; (**b**) examples of the samples with longitudinal damage before the tensile test.

Figure 4 shows the examples of the tested samples.

## 3. Results and Discussion

The obtained results provided some new understanding of the belt damage propagation and may be helpful in improving the belt condition monitoring system based on strain gauges.

### 3.1. Real-Time Monitoring of the Damaged Belt

**Figure 5:**
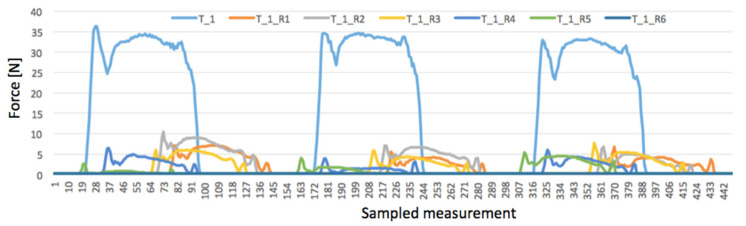
Signal registered by T1 strain gauge after cuts R1-R6 were added.

**Figure 6:**
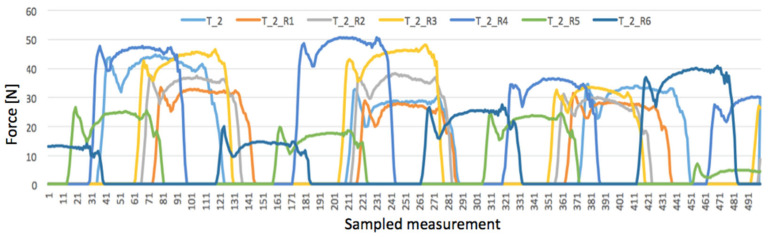
Signal registered by T2 strain gauge after cuts R1-R6 were added.

**Figure 7:**
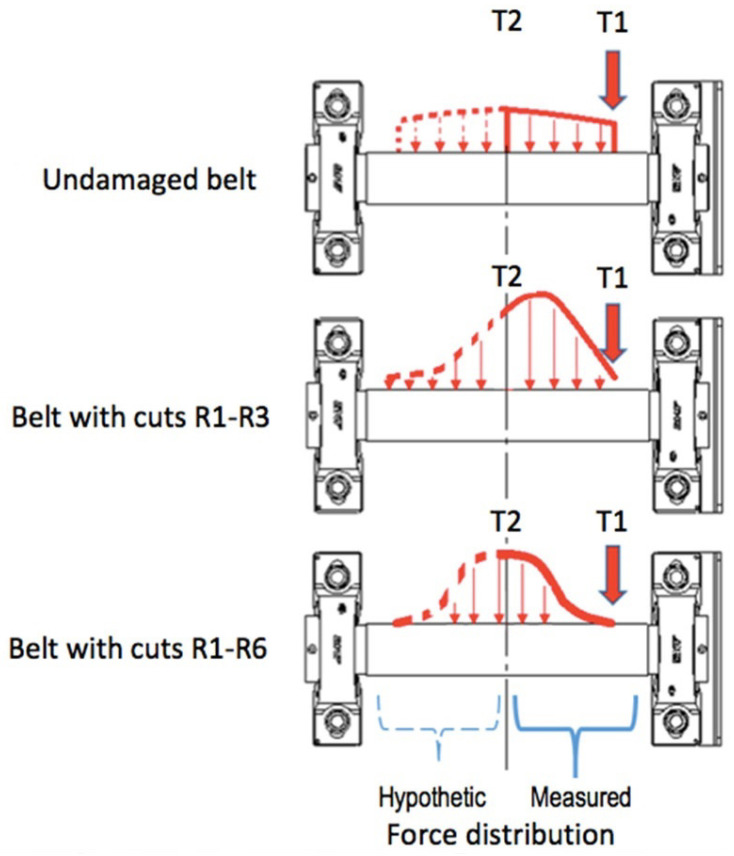
Signal registered by T2 strain gauge after cuts R1–R6 were added.

Before the measurement, the system was adjusted to make the load (35 N) equal on the strain gauges T1 and T3 in order to stabilize the inner stresses of the material. Since damage to the edge of the belt was expected, the test started with R1 damage closer to the middle of the belt (see Figure 2). The graphs in Figure 5, Figure 6 and Figure 7 represent the collections of the registered signals by the respective gauges T1, T2, T3 after the sequence of cuts from R1 to R6 was added. Since each cycle generated 10,000 results, the graphs show only four rotations of the roller (ca. 500 sampled measurements). In this way, the effect of the damage on the strain gauges’ indications is better distinguished.

Figure 5 demonstrates the impact of damage on the response of the T1 strain gauge.

The curve denoted as T_1 in Figure 5 corresponds with undamaged belt, where the T1 strain gauge under belt pressure registered forces close to 35 N. After the very first damage R1, a drastic drop in the registered forces below 10 N can be seen. Another significant decrease is seen after the R4 cut was made, in direct proximity with the T1 strain gauge. However, when all cuts R1-R6 were in place, its indication was 0.2 N.

The reaction of the middle strain gauge T2 was quite different, as illustrated in Figure 6.

First of all, it should be noted that the strain gauge T2 placed in the middle area of the roller exhibited significant differences between the registered force values, although not as large as in the case of the T1 strain gauge (Figure 5). The signal was different in each rotation, both for the undamaged belt and after each subsequent cut was made. For instance, the curve denoted as T_2_R4 (after R4 cut) reached 50 N at first roller rotation (sampled measurement 211), while at the third rotation, it was just 36.5 N (sampled measurement 361). Moreover, even the undamaged belt generated large differences in curve T_2; at the first rotation (sampled measurement 81), the strain gauge registered a signal of 44.7 N, while at the second one, it registered a signal of just 28.8 N (sample measurement 81). In fact, this phenomenon was typical for a rubber belt during conveyor’s operation, caused by complex dynamical stresses inside the material stretched between two rotating rollers [36].

On the other hand, the indications of the T2 strain gauge may seem different from the predicted or expected behavior of the system. While the pressing force on the strain gauge T1 significantly decreased after each cut, the graphs in Figure 5 show some increase in the force when the cuts from R1 to R4 were made. Only after the R5 cut did the pressing force on the middle strain gauge decrease. However, this phenomenon can be explained when considering the changes in force distribution on the roller after the damage. While the overall resultant force on the passive roller remained the same, the damaged area of the belt pressed on it with smaller force. So, it could be expected that in other areas along the passive roller, the force would increase. This can be presented schematically, as is shown in Figure 7. Unfortunately, the third strain gauge generated an excessive error during the damage test, so it is problematic to interpret its indications at this stage of the investigations. For this reason, the force distribution on the left side of the roller is presented as hypothetical.

It can be seen that the cuts R1–R3 weakened the left side of the belt, shifting maximal pressing forces on the roller and causing an increase in T2 and a decrease in T1 indications. However, cut R5 weakened the entire belt, which was detected as a drop in T2 indications by ca. 50% from 44.8 N down to 22.3 N. With the damage to the entire belt width with cuts R1–R6, the weakened belt exerted no pressure on the strain gauge T1, which indicated a signal of constantly F = 0.23 N. At the same time, the pressing force on T2 increased by 17%, which indicated that the middle area of the belt was loaded with maximal stresses.

**Figure 8:**
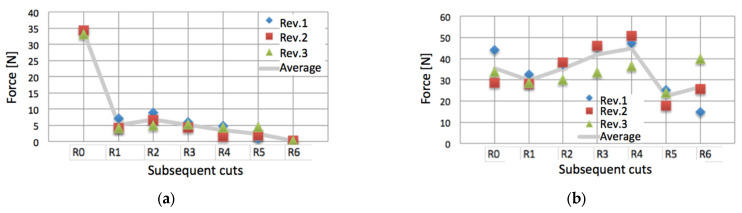
Indications of the strain gauges during three revolutions after the appearance of the subsequent cuts R1 to R6: (**a**) strain gauge T1; (**b**) strain gauge T2.

Figure 8 presents the experimental results of the changes in strain gauges’ indications for the first three roller revolutions after the appearance of subsequent cuts from R1 to R6. The line of average results indicates the overall trend.

Noteworthy, after the cut R2 was made, the shifted pressure distribution caused an increase in pressing forces on both the T1 and T2 strain gauges. Moreover, while the middle strain gauge T2 was less loaded under the undamaged belt, the damaged belt caused a several times larger load on it, as compared to the T1 gauge. In addition, the T2 gauge almost always exhibited a much larger dispersion of the indications than T1. The largest dispersion of *R_max_ =* 25 N took place after the last cut R6, when the sides of the belt did not bear any load (T1 indication was zero), demonstrating the highly instable dynamics of the inner stresses.

### 3.2. Strength Test Results

**Figure 9:**
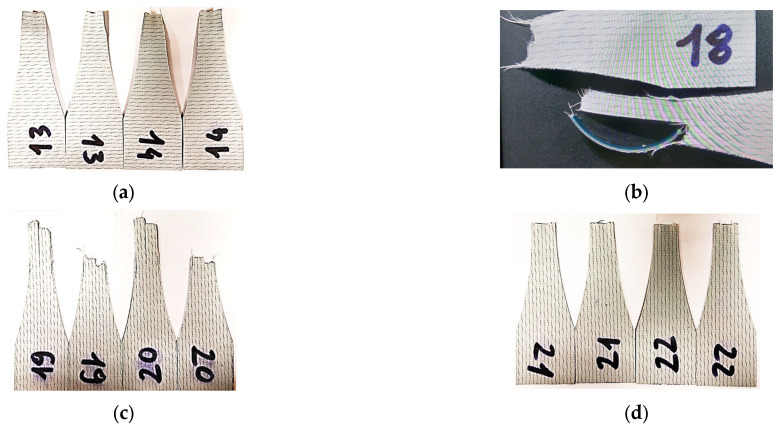
Samples after the test: (**a**) sample no. 13 and 14; (**b**) sample no. 18; (**c**) sample no. 19 and 20; (**d**) sample no. 21 and 22.

Five-element samples were used to analyze the results. Figure 9 shows sample photographs of the tested samples after rupture.

**Figure 10:**
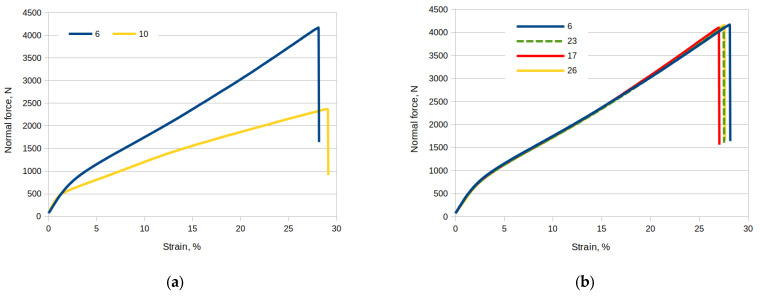
Normal force waveforms as a function of strain for selected samples: (**a**) comparison of sample no. 6 with no. 10; (**b**) comparison of samples no. 6 with no. 17, 23 and 26.

Figure 10 shows the normal force vs. strain curves. Figure 10a shows a comparison of sample no. 6 (cut along the fibers) with sample no. 10 (cut transversely to the direction of the fibers), both samples without damage. The value of the breaking force of sample no. 6 (cut along the fibers) is much larger than for sample no. 10 (cut transversely to the direction of the fibers), at almost double. Figure 10b shows a comparison of samples cut along the fibers. Sample No. 6 has no damage, sample no. 17 has a longitudinal damage of 70 mm cut through the sample, sample no. 23 has a longitudinal damage cut on the surface, and sample no. 26 has a longitudinal damage of 45 mm cut through the surface. The occurrence of damage and the size and type of damage do not affect the value of the breaking force. The occurrence of damage reduces the “strain” value slightly.

**Figure 11:**
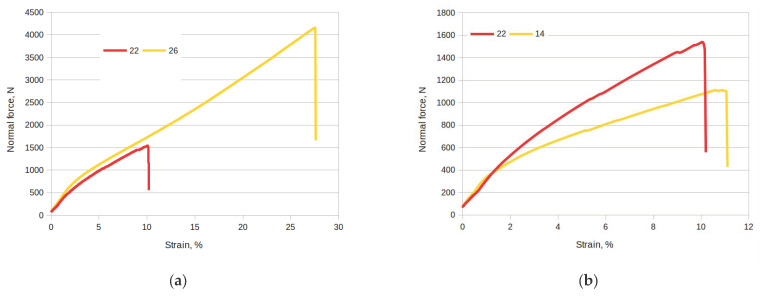
Normal force waveforms as a function of strain for selected samples: (**a**) comparison of sample no. 22 with no. 26; (**b**) comparison of sample no. 22 with no. 14.

Figure 11a compares a sample cut along the fibers with longitudinal damage (sample no. 26) with a sample with transverse damage (sample no. 22). The sample with transverse damage (sample no. 22) has a much lower breaking force than the sample cut along the fibers with longitudinal damage (sample no. 26), at almost three times the level. This also results in much lower elongation. Figure 11b compares the force curves of transversely damaged samples, where sample no. 22 is cut along the fibers, while sample no. 14 is cut across the fibers. For sample no. 22, the breaking force has a much higher value than for sample 14, but the elongation is greater for sample 14.

**Figure 12:**
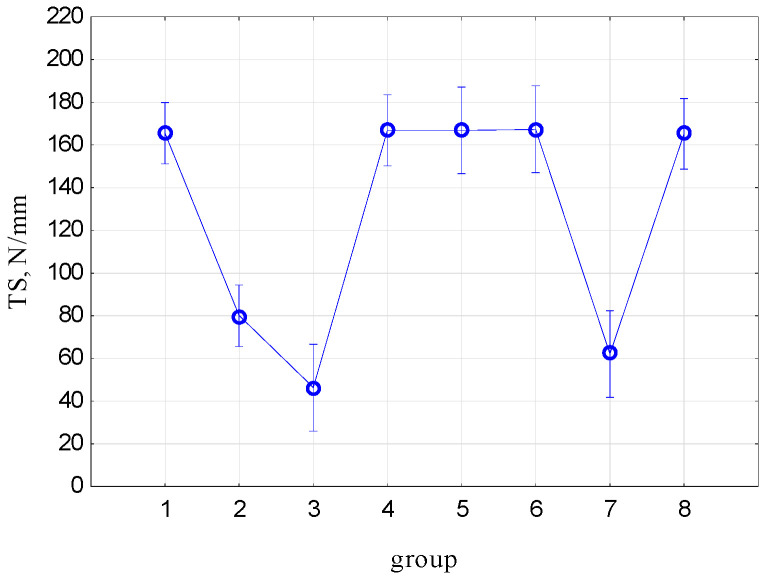
Mean tensile strength (TS) values and 95% confidence interval.

Figure 12 shows the mean tensile strength (TS) values and the 95% confidence interval. Table 2 presents the results of the ANOVA analysis with the post hoc test for intergroup relations. The type and size of damage have no significant impact on the tensile strength (TS) value for longitudinal stretching and longitudinal damage samples.

The TS value is significantly different between Group 3 and the remaining groups, except for Group 7. Group 3 and Group 7 are transversely cut samples. They have the lowest TS value and significantly, at the *p* = 0.05 level, they do not differ from each other (Group 3, transverse stretching of the belt, Group 7, longitudinal stretching of the belt). The samples in the groups without damage differ significantly at the level of *p* = 0.05 (Group 1, longitudinal stretching of the tape, Group 2, transverse stretching of the tape). After making cuts, the decisive factor for the TS value is the incision. The occurrence of longitudinal damage (cutting) does not cause a significant difference in the TS values (Groups 1, 4, 5, 6 and 8); instead, what determines the tensile strength value is whether the tape fibers have been cut. The length of the incision and its depth have no influence on TS (Groups 4, 5, 6 and 8).

**Figure 13:**
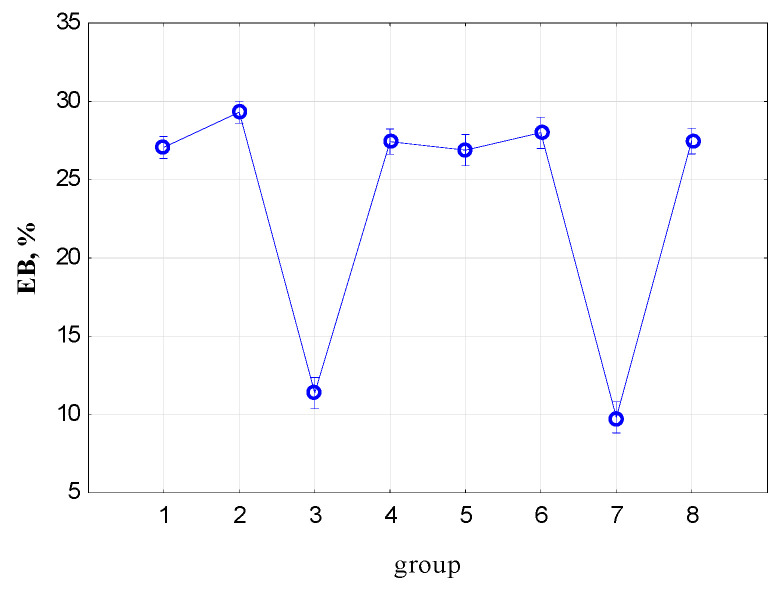
Mean values of elongation at break (EB) and 95% confidence interval.

Figure 13 shows the mean elongation at break (EB) values and the 95% confidence interval. Table 3 presents the results of the ANOVA analysis with the post hoc test for intergroup relations.

The EB value is significantly different for Groups 2, 3 and 7 from the remaining groups. Therefore, the direction of sample cutting is important for the EB value (Group 1, longitudinal stretching of the tape, Group 2, transverse stretching of the tape). Elongation at break has the greatest value for Group. 2 (samples cut across the grain). There are no significant differences between Groups 1, 4, 5, 6 and 8 at the significance level of *p* = 0.05. The occurrence of longitudinal damage (cutting) does not cause a significant difference in the EB value (Groups 1, 4, 5, 6 and 8); instead, the decisive factor for the EB value is whether the tape fibers have been cut. The length of the incision and its depth have no effect on EB (Groups 4, 5, 6 and 8).

Nominal tensile strength (NTS) was determined for typical belt operating conditions, i.e., for samples stretched longitudinally without damage:

NTS = 162.3 N/mm,(4)

Reference force, reference load (RF)—one-tenth of the nominal tensile strength in the longitudinal direction multiplied by the width of the test piece in mm:

RF = 0.1 × NTS × b = 0.1 × 162.3 × 25 = 405.75 N,(5)

**Figure 14:**
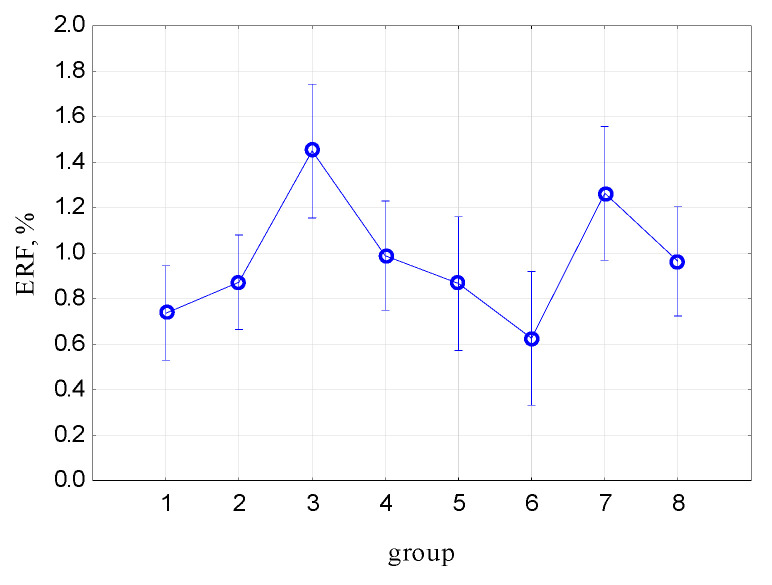
Mean values of elongation at the reference force (load) (ERF) and 95% confidence interval.

Figure 14 shows the mean values of elongation at the reference force (load) (ERF) and the 95% confidence interval. Table 4 presents the results of the ANOVA analysis with the post hoc test for intergroup relations.

For Groups 3 and 7, there are no statistically significant differences (*p* = 0.05). ERP prolongation for Group 7 shows no significant difference from Groups 4 and 5. These results confirm the results of the previous ANOVA analyses.

**Figure 15:**
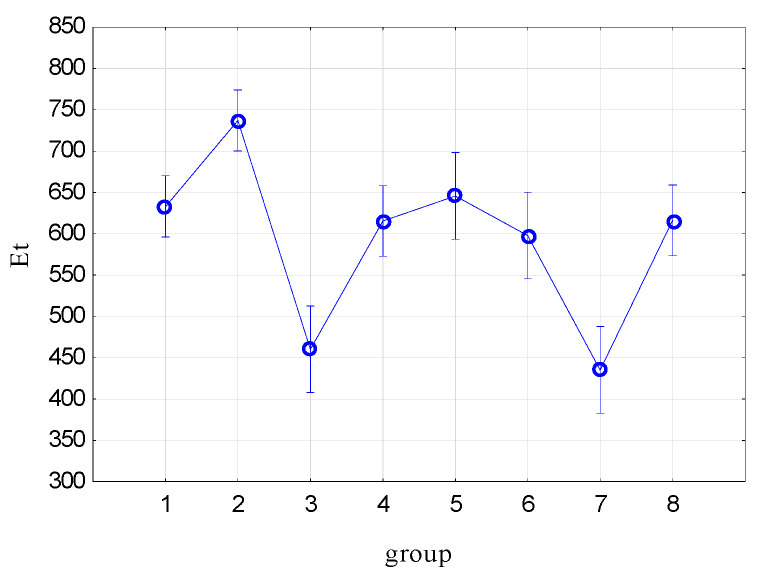
Mean tensile modulus (Et) values and 95% confidence interval.

Figure 15 shows the mean tensile modulus (Et) values and the 95% confidence interval. Table 5 presents the results of the ANOVA analysis with the post hoc test for intergroup relations.

Tensile modulus. The Et value is significantly different between Group 3 and the remaining groups, except for Group 7. Group 3 and Group 7 are transversely cut samples. They have the lowest Et value and do not differ significantly from each other (Group 3 has a transverse marking and Group 7 has a longitudinal marking). The samples of Groups 1 and 2 differed significantly when they did not have incisions; after making the incisions, the decisive factor for the Et value is the incision (Groups 4, 5, 6 and 8).

The highest average Et value is for undamaged samples with a longitudinal marking.

Analyzing the obtained data, it can be indicated that similar breaking force values were also obtained in the studies by Fedorko et al. [37] for samples with textile tape.

## 4. Conclusions

This paper presents tests of conveyor belt damage using an innovative belt condition monitoring system. In addition, strength tests of the textile–rubber belt were performed with two types of longitudinal and transverse damage, which are important for belt conveyor operators and belt manufacturers. The innovative system used to monitor the condition of the transport belt based on strain gauge systems is quite simple in design and reliable, and the results obtained are easy to interpret.

Initial experiments with belt damage monitoring revealed that the indications of the T2 strain gauge seemed different from the predicted or expected behavior of the system. Namely, the pressing force on the strain gauge T1 significantly decreased after each cut, but it increased when the first set of cuts were made. This phenomenon can be explained when considering the changes in force distribution on the roller after the damage, and it indicates the necessity to collect large amounts of data before the application of the strain gauging system for monitoring. This would require more experiments with damaged belts, which is too expensive for the present stage of the research. Moreover, the distinguishable, but complex, results indicate that it will be necessary to employ machine learning algorithms for further interpretation and decision making.

The statistical analysis of the conveyor belt strength test provided important results giving insight into the monitored material behavior when damaged. First of all, the results of the post hoc test confirm a significant difference between samples stretched longitudinally (Group 1) and those stretched transversely (Group 2). Next, the samples with longitudinal cuts had negligible mean differences compared to samples without cuts (Group 4 vs. Groups 1, 5, 6 and 8). Further, the highest average Et values were exhibited by the undamaged samples with a longitudinal marking.

These results provide evidence for the largely differentiated impact of damage on the belt’s behavior, which is nevertheless distinguishable and identifiable. In future research, damage will be grouped and its effect on strain gauge signals will be classified using artificial intelligence and big data analytical methods.

## Figures and Tables

**Table 1:** Grouping of research samples.

Group	Description
1	No damage, longitudinal stretching
2	No damage, transverse stretching
3	Transverse damage 10 mm; transverse stretching
4	Longitudinal damage (45 mm × 1 mm); longitudinal stretching
5	Longitudinal damage (70 mm × 2 mm); longitudinal stretching
6	Longitudinal damage (50 mm × 2 mm); longitudinal stretching
7	Transverse damage 10 mm; longitudinal stretching
8	Longitudinal damage (50 mm × 1.5 mm); longitudinal stretching

**Table 2:** Results of the ANOVA analysis for tensile strength (TS) with the post hoc test for intergroup relations.

Group	1	2	3	4	5	6	7
2	0.000000						
3	0.000000	0.011551					
4	0.897573	0.000001	0.000000				
5	0.910496	0.000003	0.000000	0.999149			
6	0.873358	0.000003	0.000000	0.965263	0.967512		
7	0.000000	0.144869	0.258546	0.000001	0.000002	0.000002	
8	0.978059	0.000001	0.000000	0.883826	0.896852	0.861708	0.000001

**Table 3:** Results of the ANOVA analysis for elongation at break (EB) with the post hoc test for intergroup relations.

Group	1	2	3	4	5	6	7
2	0.000272						
3	0.000000	0.000000					
4	0.474172	0.002251	0.000000				
5	0.770405	0.000834	0.000000	0.384542			
6	0.122127	0.038026	0.000000	0.360354	0.114587		
7	0.000000	0.000000	0.033454	0.000000	0.000000	0.000000	
8	0.432973	0.002595	0.000000	0.947378	0.354430	0.390807	0.000000

**Table 4:** Results of the ANOVA analysis for elongation at the reference force (load) (ERF) with the post hoc test for intergroup relations.

Group	1	2	3	4	5	6	7
2	0.339345						
3	0.000814	0.003997					
4	0.109929	0.442152	0.020974				
5	0.451887	0.973482	0.009421	0.499108			
6	0.521073	0.164769	0.000813	0.059310	0.235281		
7	0.007278	0.035333	0.353113	0.143941	0.059965	0.005403	
8	0.145757	0.542307	0.015977	0.878515	0.587888	0.076392	0.113715

**Table 5:** Results of the ANOVA analysis for tensile modulus (Et) with the post hoc test for intergroup relations.

Group	1	2	3	4	5	6	7
2	0.000792						
3	0.000048	0.000000					
4	0.515098	0.000403	0.000224				
5	0.677636	0.008583	0.000098	0.352620			
6	0.259681	0.000373	0.001351	0.587164	0.187718		
7	0.000012	0.000000	0.480120	0.000053	0.000028	0.000333	
8	0.532818	0.000426	0.000215	0.979235	0.364251	0.571503	0.000051

## Data Availability

Data will be made available upon request.
